# Supplementary material for: Efficacy of Pilates on Pain, Functional Disorders and Quality of Life in Patients with Chronic Low Back Pain: A Systematic Review and Meta-Analysis
Source: Int J Environ Res Public Health. 2023 Feb 6;20(4):2850. doi: 10.3390/ijerph20042850 (PMC9956295; doi:10.3390/ijerph20042850)
Supplement: Supplementary file 1 [file ijerph-20-02850-s001.zip › ijerph-2129937-supplementary.pdf]

Study or Subgroup	Experimental Mean	Experimental SD	Experimental Total	Control Mean	Control SD	Control Total	Weight	Std. Mean Difference IV, Random, 95% CI
Cruz-Díaz D2016	4.17	1.36	53	6.21	1.74	48	17.7%	-1.30 [-1.74, -0.87]
Mazloum V2018	3	0.9	16	6.9	1.6	16	13.2%	-2.93 [-3.96, -1.90]
Miyamoto G C2013	4.5	2.2	43	5.3	2.3	43	17.7%	-0.35 [-0.78, 0.07]
Natour J2015	4.2	2.78	30	5.83	2.88	30	17.2%	-0.57 [-1.09, -0.05]
Wajswelner H2012	2.5	1.8	44	2.2	1.7	43	17.8%	0.17 [-0.25, 0.59]
Yang C Y2021	5.07	2.16	20	4.42	2.65	19	16.4%	0.26 [-0.37, 0.90]
**Total (95% CI)**			**206**			**199**	**100.0%**	**-0.70 [-1.38, -0.03]**

Heterogeneity: Tau² = 0.62; Chi² = 50.45, df = 5 (P < 0.00001); I² = 90%
Test for overall effect: Z = 2.04 (P = 0.04)

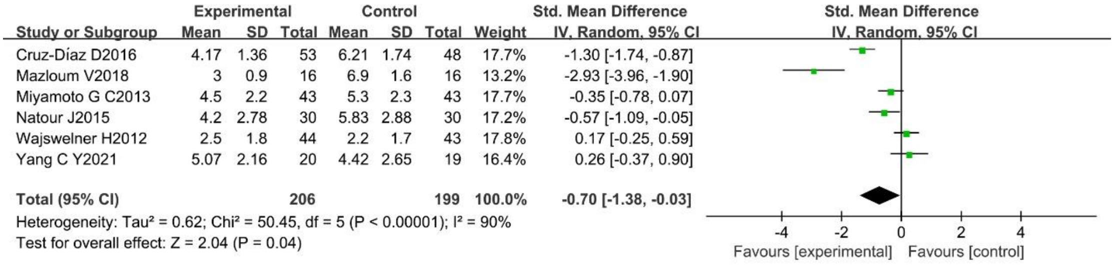

Supplementary Figure S1. Meta-analysis of the effect of Pilates on Pain Scale follow-up.

Study or Subgroup	Experimental Mean	Experimental SD	Experimental Total	Control Mean	Control SD	Control Total	Weight	Mean Difference IV, Random, 95% CI
Cruz-Díaz D2016	10.02	2.27	53	19.98	3.58	48	52.6%	-9.96 [-11.14, -8.78]
Mazloum V2018	22.9	3.6	16	26.6	5	16	47.4%	-3.70 [-6.72, -0.68]
**Total (95% CI)**			**69**			**64**	**100.0%**	**-6.99 [-13.12, -0.86]**

Heterogeneity: Tau² = 18.23; Chi² = 14.32, df = 1 (P = 0.0002); I² = 93%
Test for overall effect: Z = 2.24 (P = 0.03)

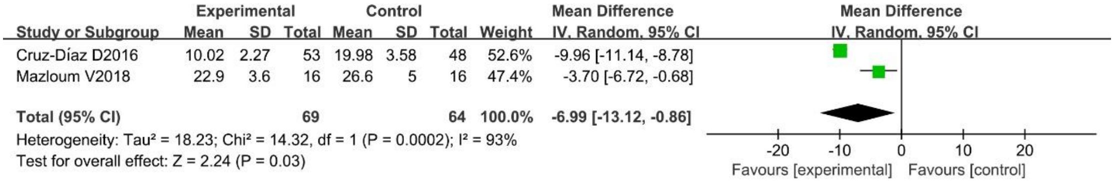

Supplementary Figure S2. Meta-analysis of the effect of Pilates on ODI follow-up.

Study or Subgroup	Experimental Mean	Experimental SD	Experimental Total	Control Mean	Control SD	Control Total	Weight	Mean Difference IV, Fixed, 95% CI
Miyamoto G C2013	4.5	4.5	43	6.7	5.6	43	53.0%	-2.20 [-4.35, -0.05]
Natour J2015	7.04	5.44	30	10.66	6.23	30	27.9%	-3.62 [-6.58, -0.66]
Yang C Y2021	6.5	5.7	20	5.4	5.7	19	19.1%	1.10 [-2.48, 4.68]
**Total (95% CI)**			**93**			**92**	**100.0%**	**-1.97 [-3.53, -0.40]**

Heterogeneity: Chi² = 4.06, df = 2 (P = 0.13); I² = 51%
Test for overall effect: Z = 2.47 (P = 0.01)

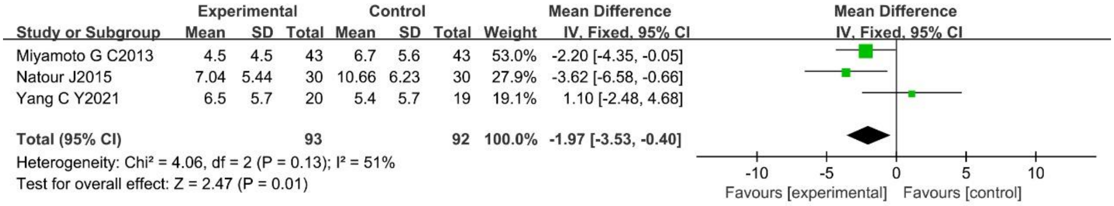

Supplementary Figure S3. Meta-analysis of the effect of Pilates on RMDQ follow-up.

Study or Subgroup	Experimental Mean	Experimental SD	Experimental Total	Control Mean	Control SD	Control Total	Weight	Mean Difference IV, Random, 95% CI
Natour J2015	65.41	28.01	30	59.58	19.04	30	43.6%	5.83 [-6.29, 17.95]
Wajswelner H2012	75.6	21.1	44	83.3	14.1	43	56.4%	-7.70 [-15.23, -0.17]
**Total (95% CI)**			**74**			**73**	**100.0%**	**-1.80 [-14.95, 11.35]**

Heterogeneity: Tau² = 65.04; Chi² = 3.46, df = 1 (P = 0.06); I² = 71%
Test for overall effect: Z = 0.27 (P = 0.79)

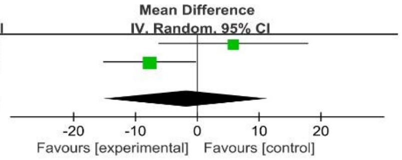

A-Physical Function

Study or Subgroup	Experimental Mean	Experimental SD	Experimental Total	Control Mean	Control SD	Control Total	Weight	Mean Difference IV, Random, 95% CI
Natour J2015	56.37	44.77	30	39.95	31.27	30	41.1%	16.42 [-3.12, 35.96]
Wajswelner H2012	82	18.1	44	87.5	17	43	58.9%	-5.50 [-12.88, 1.88]
**Total (95% CI)**			**74**			**73**	**100.0%**	**3.52 [-17.62, 24.66]**

Heterogeneity: Tau² = 183.46; Chi² = 4.23, df = 1 (P = 0.04); I² = 76%
Test for overall effect: Z = 0.33 (P = 0.74)

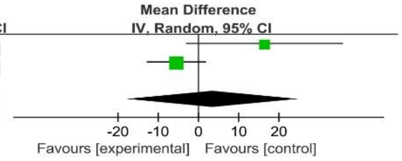

B-Role Physical

Study or Subgroup	Experimental Mean	Experimental SD	Experimental Total	Control Mean	Control SD	Control Total	Weight	Mean Difference IV, Random, 95% CI
Natour J2015	52.16	24.57	30	43.87	29.09	30	42.8%	8.29 [-5.34, 21.92]
Wajswelner H2012	73.5	18.9	44	80.7	15.6	43	57.2%	-7.20 [-14.48, 0.08]
**Total (95% CI)**			**74**			**73**	**100.0%**	**-0.57 [-15.59, 14.45]**

Heterogeneity: Tau² = 88.92; Chi² = 3.86, df = 1 (P = 0.05); I² = 74%
Test for overall effect: Z = 0.07 (P = 0.94)

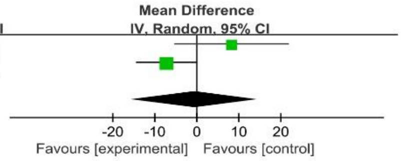

C-Bodily Pain

Study or Subgroup	Experimental Mean	Experimental SD	Experimental Total	Control Mean	Control SD	Control Total	Weight	Mean Difference IV, Fixed, 95% CI
Natour J2015	65.2	22.15	30	62.08	21.08	30	32.5%	3.12 [-7.82, 14.06]
Wajswelner H2012	74.6	20.2	44	75.7	15.7	43	67.5%	-1.10 [-8.69, 6.49]
**Total (95% CI)**			**74**			**73**	**100.0%**	**0.27 [-5.97, 6.51]**

Heterogeneity: Chi² = 0.39, df = 1 (P = 0.53); I² = 0%
Test for overall effect: Z = 0.09 (P = 0.93)

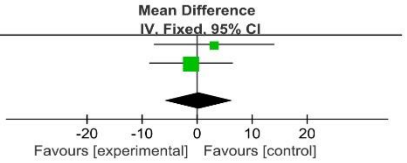

D-General Health

Study or Subgroup	Experimental Mean	Experimental SD	Experimental Total	Control Mean	Control SD	Control Total	Weight	Mean Difference IV, Fixed, 95% CI
Natour J2015	60.29	23.41	30	55	21.71	30	32.6%	5.29 [-6.13, 16.71]
Wajswelner H2012	62.7	18.4	44	62.1	19.4	43	67.4%	0.60 [-7.35, 8.55]
**Total (95% CI)**			**74**			**73**	**100.0%**	**2.13 [-4.40, 8.65]**

Heterogeneity: Chi² = 0.44, df = 1 (P = 0.51); I² = 0%
Test for overall effect: Z = 0.64 (P = 0.52)

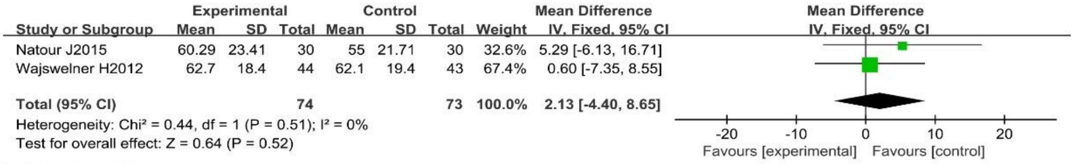

E-Vitality

Study or Subgroup	Experimental Mean	Experimental SD	Experimental Total	Control Mean	Control SD	Control Total	Weight	Mean Difference IV, Fixed, 95% CI
Wajswelner H2012	88.8	18.1	44	86.3	20.1	43	67.7%	2.50 [-5.54, 10.54]
Natour J2015	86.04	22.75	30	80.41	23.3	30	32.3%	5.63 [-6.02, 17.28]
**Total (95% CI)**			**74**			**73**	**100.0%**	**3.51 [-3.11, 10.13]**

Heterogeneity: Chi² = 0.19, df = 1 (P = 0.66); I² = 0%
Test for overall effect: Z = 1.04 (P = 0.30)

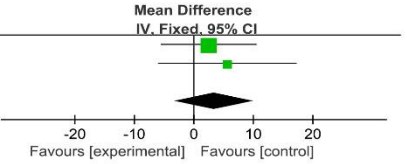

F-Social Functioning

Study or Subgroup	Experimental Mean	Experimental SD	Experimental Total	Control Mean	Control SD	Control Total	Weight	Mean Difference IV, Random, 95% CI
Natour J2015	56.37	44.77	30	39.95	31.27	30	41.1%	16.42 [-3.12, 35.96]
Wajswelner H2012	82	18.1	44	87.5	17	43	58.9%	-5.50 [-12.88, 1.88]
**Total (95% CI)**			**74**			**73**	**100.0%**	**3.52 [-17.62, 24.66]**

Heterogeneity: Tau² = 183.46; Chi² = 4.23, df = 1 (P = 0.04); I² = 76%
Test for overall effect: Z = 0.33 (P = 0.74)

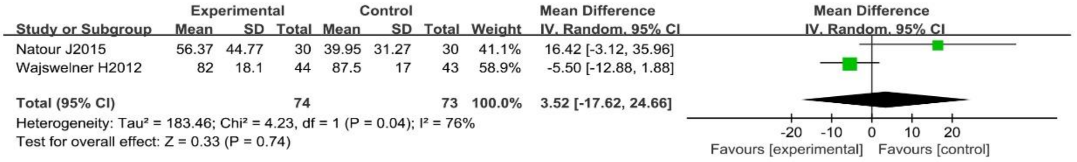

G-Role Emotion

Study or Subgroup	Experimental Mean	Experimental SD	Experimental Total	Control Mean	Control SD	Control Total	Weight	Mean Difference IV, Fixed, 95% CI
Wajswelner H2012	79.5	13.2	44	82	12.1	43	82.2%	-2.50 [-7.82, 2.82]
Natour J2015	67.9	22.05	30	65.33	23.07	30	17.8%	2.57 [-8.85, 13.99]
**Total (95% CI)**			**74**			**73**	**100.0%**	**-1.60 [-6.42, 3.23]**

Heterogeneity: Chi² = 0.62, df = 1 (P = 0.43); I² = 0%
Test for overall effect: Z = 0.65 (P = 0.52)

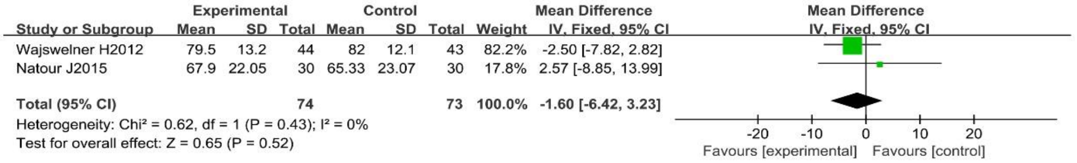

H-Mental Health

Supplementary Figure S4. Meta-analysis of the effect of Pilates on SF-36 follow-up.

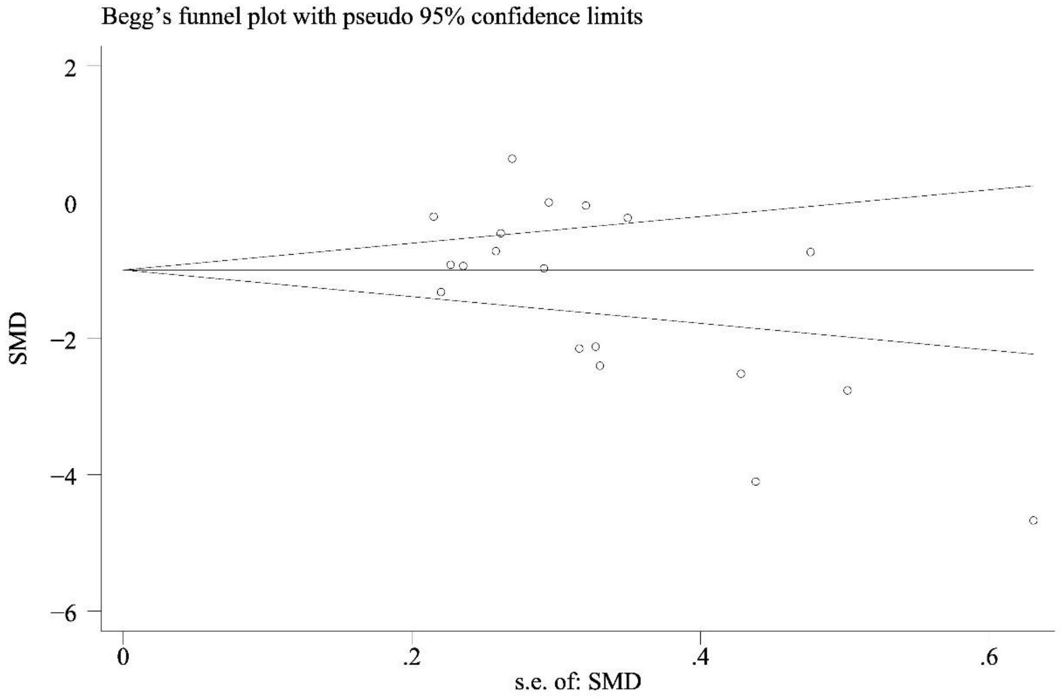

Supplementary Figure S5. Pain Scale publication bias graph of the included RCTs.

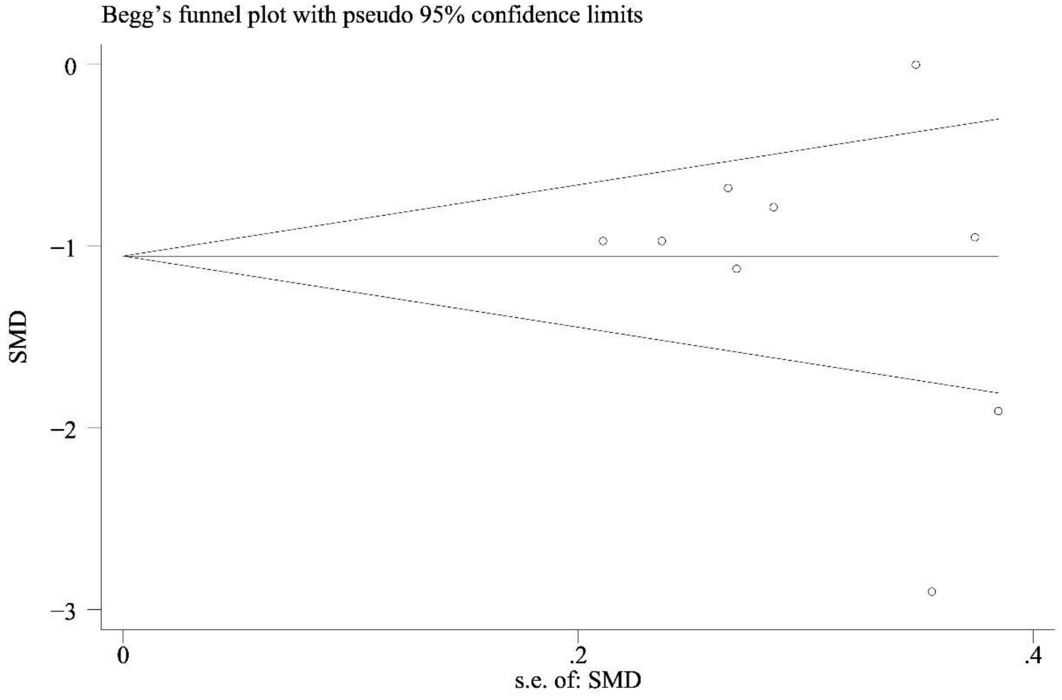

Supplementary Figure S6. ODI publication bias graph of the included RCTs.

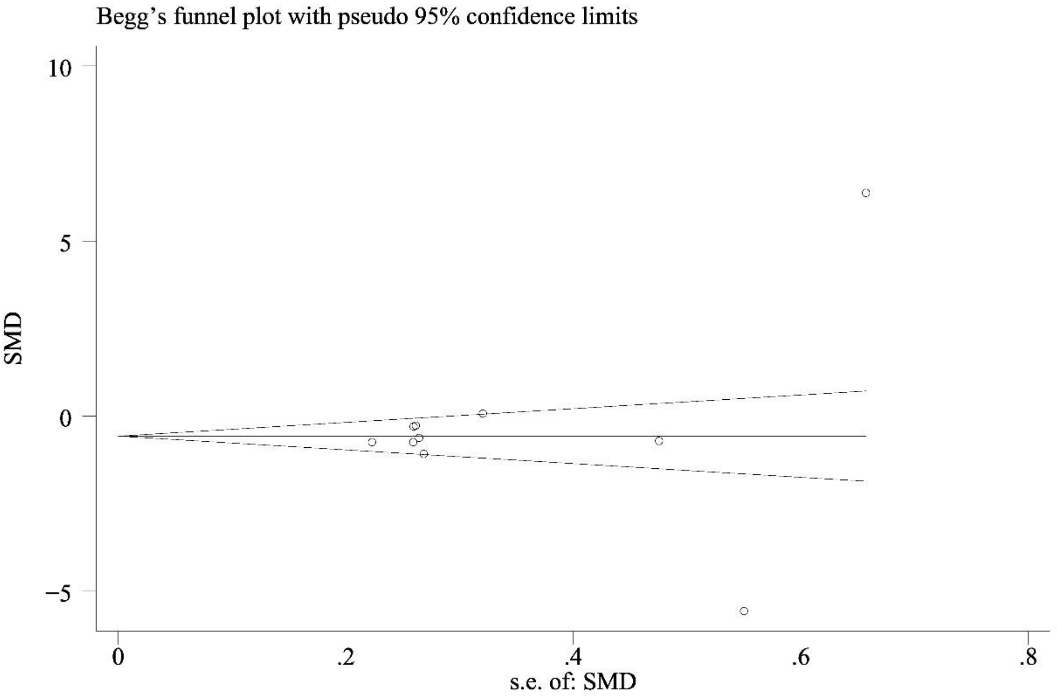

Supplementary Figure S7. RMDQ publication bias graph of the included RCTs.

Supplementary Table S1. Search strategy for each database.

Search engine	Search	Search query	Date of search
CNKI/VIP/CBM	#1	主题 (Topic) =普拉提(Pilates) OR 普拉提训练(Pilates training)	20-November-22
	#2	主题 (Topic) =腰痛(back pain/ low back pain) OR 非特异性腰痛(nonspecific low back pain) OR 慢性腰痛(chronic low back pain) OR 慢性非特异性腰痛(chronic nonspecific low back pain)	
	#3	#1 AND #2	
PubMed	#1	Pilates [Title/Abstract]	20-November-22
	#2	Pilates training [Title/Abstract]	
	#3	#1 OR #2	
	#4	low back pain [Title/Abstract]	
	#5	back pain [Title/Abstract]	
	#6	low back ache [Title/Abstract]	
	#7	chronic low back pain [Title/Abstract]	
	#8	nonspecific low back pain[Title/Abstract]	
	#9	chronic nonspecific low back pain [Title/Abstract]	

	#10	chronic nonspecific lumbago [Title/Abstract]	
	#11	chronic nonspecific lower back pain [Title/Abstract]	
	#12	chronic nonspecific lumbar pain[Title/Abstract]	
	#13	non-specific lower back pain [Title/Abstract]	
	#14	#4 OR #5 OR #6 OR #7 OR #8 OR #9 OR #10 OR #11 OR #12 OR #13	
	#15	#3 AND #14	
Web of Science	#1	TS = (‘Pilates’ OR ‘Pilates training’)	20-November-22
	#2	TS = (‘low back pain’ OR ‘back pain’ OR ‘low back ache’ OR ‘chronic low back pain’ OR ‘nonspecific low back pain ’ OR ‘nonspecific low back pain’ OR ‘chronic nonspecific low back pain’ OR ‘chronic nonspecific lumbago’ OR ‘chronic nonspecific lower back pain’ OR ‘chronic nonspecific lumbar pain’ OR ‘non-specific lower back pain’)	
	#3	#1 AND #2<br>Databases = SCI-EXPANDED, SSCI, A&HCI, CPCI-S, CPCI-SSH, ESCI	
Embase	#1	‘Pilates’ OR ‘Pilates training’	20-November-22
	#2	‘low back pain’ OR ‘back pain’ OR ‘low back ache’ OR ‘chronic low back pain’ OR ‘nonspecific low back pain ’ OR ‘nonspecific low back pain’ OR ‘chronic nonspecific low back pain’ OR ‘chronic nonspecific lumbago’ OR ‘chronic nonspecific lower back pain’ OR ‘chronic nonspecific lumbar pain’ OR ‘non-specific lower back pain’	
	#3	#1 AND #2	
EBSCO	S1	Pilates OR Pilates training	20-November-22
	S2	low back pain OR back pain OR low back ache OR chronic low back pain OR nonspecific low back pain OR nonspecific low back pain OR chronic nonspecific low back pain OR chronic nonspecific lumbago OR chronic nonspecific lower back pain OR chronic nonspecific lumbar pain OR non-specific lower back pain	
	S3	S1 AND S2	

Supplementary Table S2. Jadad scale score of included RCTs.

Reference	Generation of random sequences	Randomization concealment	Blind method	Withdrawal	Total score	Level
Gladwell V.2005	1	0	2	1	4	H
Rydeard R.2006	2	1	0	1	4	H
Wajswelner H.2012	2	2	2	1	7	H
Miyamoto G C.2013	2	2	2	1	7	H
Notarnicola A.2014	0	0	0	1	1	L
Mostagi F Q.2015	2	2	2	1	7	H
Natour J.2015	2	2	2	1	7	H
Akodu A.2016	1	0	2	0	3	L
Cruz-Díaz D.2016	2	2	2	1	7	H
Cruz-Díaz D.2017	2	1	2	1	6	H
Lopes S.2017	2	1	1	1	5	H
Mazloum V.2018	1	0	2	1	4	H
Ying Z.2019	2	1	0	1	4	H
Minghui L.2019	1	1	0	1	3	L
Baskan Ö.2021	2	1	2	1	6	H
Batıbay S.2021	1	1	2	0	4	H
Yang C.2021	2	1	2	2	6	H
Fei W.2022	1	1	0	1	3	L
Manman M.2022	1	1	0	1	3	L

Table notes: The numbers are the scores of each item. The higher the score, the higher the quality. "Total score" represents the sum of the scores of each item. "H" means high quality. "L" means low quality.

Supplementary Table S3. The pooled results of sensitivity analyses [MD (95% CI)].

Indicators	Minimum estimate	Maximum estimate	Overall result
Pain Scale	-1.412(-1.922, -0.903)	-1.272(-1.772, -0.772)	-1.336(-1.829, -0.842)
ODI	-1.250(-1.691, -0.810)	-0.913(-1.207, -0.619)	-1.124(-1.572, -0.675)
RMDQ	-1.013(-1.659, -0.366)	0.073(-0.674,0.821)	-0.405(-1.311, 0.502)
